# Getting it right before transplantation: example of a stem cell model with regenerative potential for the CNS

**DOI:** 10.3389/fcell.2014.00036

**Published:** 2014-08-13

**Authors:** Cedric Viero, Oksana Forostyak, Eva Sykova, Govindan Dayanithi

**Affiliations:** ^1^Experimental and Clinical Pharmacology and Toxicology, Medical Faculty, Saarland UniversityHomburg, Germany; ^2^Department of Molecular Neurophysiology, Institute of Experimental Medicine, Academy of Sciences of the Czech RepublicPrague, Czech Republic; ^3^Department of Neuroscience, Second Medical Faculty, Charles UniversityPrague, Czech Republic; ^4^Department of Neuroscience, Institute of Experimental Medicine, Academy of Sciences of the Czech RepublicPrague, Czech Republic; ^5^Institut National de la Santé et de la Recherche Médicale, Unité de Recherche U710, Université Montpellier 2Montpellier, France; ^6^Ecole Pratique des Hautes EtudesParis, France

**Keywords:** human embryonic stem cell, neural precursors, neurodegenerative diseases, spinal cord, immortalized stem cell lines, calcium signaling, ion channels

## Abstract

The burden of neurodegenerative disorders in an aging population has become a challenge for the modern world. While the biomarkers available and the methods of diagnosis have improved to detect the onset of these diseases at early stages, the question of adapted and efficient therapies is still a major issue. The prospect of replacing the loss of functional neural cells remains an attractive but still audacious approach. A huge progress has been made in the generation of neurons derived from human stem cell lines and transplantation assays are tested in animals for a wide range of pathologies of the central nervous system. Here we take one step back and examine neuronal differentiation and the characterization of neural progenitors derived from human embryonic stem cells. We gather results from our previous studies and present a cell model that was successfully used in functional analyses and engraftment experiments. These neuronal precursors exhibit spontaneous and evoked activity, indicating that their electrophysiological and calcium handling properties are similar to those of matured neurons. Hence this summarized information will serve as a basis to design better stem cell-based therapies to improve neural regeneration.

## Introduction

The present general interest in stem cell research results from the promising dual perspective proposed by this advancing technology: on the one hand, it offers a source of *in vitro* models for human cells that can mimic pathological states, and, on the other hand, a possible therapeutic tool to induce cellular (and tissue) regeneration. In both cases, the public organizations, currently trying to generate reliable stem cell registries, have to face an increasing number of cell lines from all over the world. Human embryonic stem cell (hESC) lines which can be commercialized and used as candidates for cell therapies need to fulfill a number of eligibility requirements starting from the moment of tissue donation which are regulated by the NIH guidelines on human stem cell research and by the U.S. Food and Drug Administration (FDA). For example, the donors should fulfill the eligibility requirements for tissue donors, including a number of tests for infectious agents and diseases, which in many cases can be hardly achieved due to some ethical and legal issues (for details, see Jonlin, 2014).

The European hESC registry lists over 700 hESC lines and 52 human induced pluripotent stem cell (hiPSC) lines. The International Stem Cell Registry, hosted by the University of Massachusetts Worcester Campus, documents 1303 records for hESCs and 281 records for hiPSCs. In addition, the NIH hESC registry reports 261 cell lines having a registration number (see also Adewumi et al., 2007). Beside the need for a unified international system to register the plethora of human stem cell lines available at this date and in the future, these numbers highlight the substantial variety of sources of starting material, and of methods for cultivation, derivation and subculture. There is not only multiplicity in the compounds and protocols used in stem cell research, but also a disparity (leading sometimes to controversies) in the experimental approaches to characterize these novel cell lines.

Taking into account these real challenges as well as being aware that not all of them can be currently solved, we propose the basic steps necessary to create and select a putative human stem cell line that could be employed for neural regeneration, especially with regard to transplantation assays.

After injection *in vivo*, the cells in question should display the ability to survive, proliferate, migrate, differentiate and maintain a neural phenotype; conversely undifferentiated stem cells would form tumors comprising of cells from all three embryonic germ layers.

Many past and present studies have investigated the differentiation of hESCs into the main neuronal and glial subtypes: dopaminergic, GABA(γ-aminobutyric acid)ergic, glutamatergic neurons, astrocytes, and oligodendrocytes. Whilst possible, the transplantation of terminally differentiated cells is difficult and not ideal. Considering this the best option for cell therapy remains the use of neural precursors (NPs); however, this method is not without limitations as it is coupled with the risk of tumor formation. Currently, there are not many reports available on tumor prevention mechanisms. Several studies have demonstrated the role of specific microRNAs (e.g., miR-302) in the control of pluripotency (Eini et al., 2013). These findings, together with future studies, may provide a means to control hESC tumorigenicity and improve the safety of hESC with regard to therapeutic use (Eini et al., 2013; Lin and Ying, 2013; Shah and Allegrucci, 2013).

## Choice of a cell line: preliminary tests

The hESCs are prone to instability and variations; therefore their culture is a challenging process. To assure the good quality of hESCs, they have to meet the following critical criteria: (i) purity (absence of any microbial contamination), (ii) identity (corresponding to the correct profile of the cells), and (iii) stability (the genotype and phenotype remain stable during passaging *in vitro*). Particularly mycoplasma contamination may inhibit cell growth, affect cell metabolism, and increase sensitivity to inducers of apoptosis (Marx, 2014). Mycoplasmas are much smaller than typical bacteria and the lack of cell walls makes these organisms resistant to many antibiotics. There are a number of techniques that are applied to mycoplasma detection. For routine testing direct PCR or Hoechst 33258 staining have been used.

Using the CCTL14 hESC line as an example (Kozubenko et al., 2010), after making sure that the cells have the expected karyotype, it is required to analyze the expression of several pluripotent and neuroectodermal markers in undifferentiated hESCs before starting the differentiation procedure. The undifferentiated hESCs should express high levels of pluripotent markers such as OCT-3/4 and Nanog, which are nuclear-localized transcription factors, and SSEA-4, a surface antigen. Immunocytochemical results demonstrated single CCTL14 cells in the colonies to be positive for SSEA-1, whilst no positive staining was found for NCAM, NF70, nestin, or β-III tubulin.

Single cell suspensions were labeled with antibodies directed against Nanog, SSEA-4, SSEA-1, TRA-1-60, CD24, CD133, CD56 (NCAM), β-III-tubulin, NF70, nestin, CD271 (NGFR), CD29, and HLA-ABC. The expression profiles were categorized as follows, based on the percentage of positive cells: 0–5% negative, 6–39% low, 40–79% moderate, and 80–100% high. Flow cytometric analysis revealed high levels of Nanog and SSEA-4 expression, moderate levels of TRA-1-60, CD24, and β-III tubulin expression, and low levels of SSEA-1, nestin and HLA-ABC expression. Undifferentiated hESCs were negative for several neuroectodermal markers: CD133, NCAM, NF70, NGFR, and CD29.

**Figure 1:**
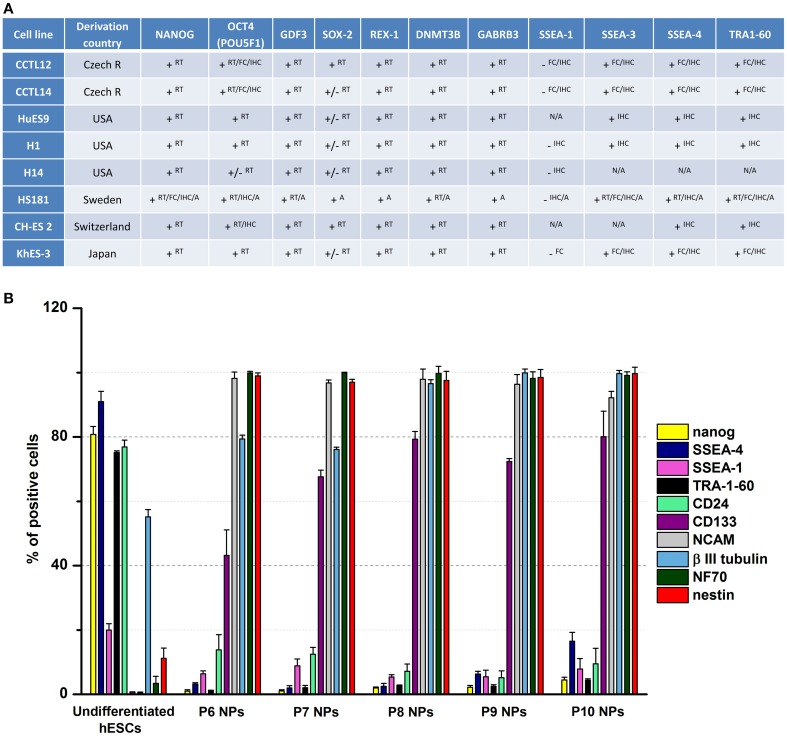
**Marker expression analysis. (A)** This table summarizes the expression of characteristic markers in different lines of human embryonic stem cells. The data were taken from the European Human Pluripotent Stem Cell Registry. ^RT^, RT-PCR; ^FC^, Flow cytometry; ^IHC^, Immunocytochemistry/Immunohistochemistry; ^A^, Array; ^+^, detected; ^−^, not detected; ^+/−^, weak signal; ^N/A^, not applicable. **(B)** Fluorescence-activated cell sorting profiles of pluripotent and neural markers in undifferentiated hESCs and hESC-derived NPs (P6–P10) during long-term propagation *in vitro*. The predifferentiation of hESCs led to the downregulation of pluripotent markers (Nanog, SSEA-4, SSEA-1, TRA-1-60, and CD24) and upregulation of neural markers (CD133, NCAM, β-III tubulin, NF70, and nestin). Data presented in Kozubenko et al. (2010) and Forostyak et al. (2013) were pooled and revised to prepare this figure.

The rationale behind this analysis is to show how undifferentiated cells are positive for several pluripotent markers, but do not express typical neural markers (Figure 1A).

## Differentiation into neural precursors

The production of NPs can currently be obtained via three methods: (i) using a co-culture with a stromal cell line or stromal feeder-based differentiation (Kawasaki et al., 2000; Vazin et al., 2008; Kim and Park, 2011), (ii) using the recently more conventional formation of embryoid bodies (EBs) (Lee et al., 2000), which is also widely employed to differentiate stem cells into cardiomyocytes (Burridge et al., 2007), or (iii) using the monolayer culture system (Reubinoff et al., 2001; Chambers et al., 2009; Li et al., 2011). The main advantage of the adherent monolayer protocol over the EB protocol is based on the rapidity of the time for neural induction (6–9 vs. 16–19 days). However neural rosettes cannot easily be distinguished and isolated with the monolayer culture system.

It is worth mentioning at this point that not all neurospheres are produced from a neural stem cell and can therefore be generated from neural progenitors; this may be an issue in attempts to amplify cultures enriched in neural stem cells. For this particular reason, the Neural Colony-Forming Cell Assay was developed based on the capacity of single cells to generate colonies of various sizes depending on their level of differentiation (see Louis et al., 2008), which thus provides a reliable tool to quantify neural stem cells in comparison to progenitor cells. It is commercially available for neural colonies derived from mouse embryonic central nervous system (CNS) tissue.

For the derivation of neuroectodermal progenitors from the CCTL14 line of hESCs an effective differentiation protocol was developed based on the formation, propagation and expansion of sphere cultures. After 14 days of induction, the NPs were further passaged in a monolayer (Kozubenko et al., 2010).

The use of fetal calf serum and mouse feeder cells potentially exposes the human cells to unknown pathogens (viruses and prions), which might be transmitted between species. Both serum and feeder cells may also contain many undefined components that may influence the differentiation of hESCs. The elimination of these factors from the culture process is one of the major challenges in the development of hESC culture systems and with regard to their further application in translational medicine. The propagation of hESCs, using mammalian- or human-derived extracellular matrix and conditioned medium from feeder cells, may represent a potential approach to solve these issues (Datta et al., 2013).

## Characterization of the hESC-derived neural precursors

To assess the success of the differentiation procedure in terms of neuronal specification and corresponding yield, we used a combination of assays based on the expression of molecular markers and cell function.

### Expression of molecular markers

After 5 and 8 passages, hESC-derived NPs underwent a flow cytometric analysis using antibodies against: CD29, CD271 (neural growth factor receptor), HLA-ABC, CD15 (SSEA-1), CD56 (neural cell adhesion molecule), CD24, CD133/1, CD133/2, Nanog, TRA-1-60, SSEA-4, nestin, neurofilament 70 kDa (NF70), and NCAM (Figure 1B). In parallel real-time PCR was performed employing primers for: nestin, Nanog, Oct4, Cripto, AFP, Sox2, SSEA-1, α-actin, VGEFR, Nodal, GATA4, and Prom1 (CD133).

To confirm the presence of NPs and the level of differentiation, immunocytochemistry was employed with antibodies targeted against synaptophysin, β-III tubulin, neurofilament 160 kDa (NF-160), GABA, and glutamate.

In an additional immunostaining investigation, we further detected the following neural markers: NeuN, β-III tubulin, GFAP, S100, OLIG, GS (Forostyak et al., 2013). Moreover we provided evidence of the presence of important Ca^2+^-sensitive channels: L-type voltage-gated Ca^2+^ channels (VGCC), P/Q-type VGCC, Ryanodine receptor Ca^2+^ release channels (RyR) type 1, RyR2 (in only a few cells), and RyR3. Finally we could detect the expression of the purinergic receptors P2X_2_, P2X_3_, and P2X_7_, which work as substrates for the fast excitatory neurotransmitter ATP.

### Cell function assay

**Figure 2:**
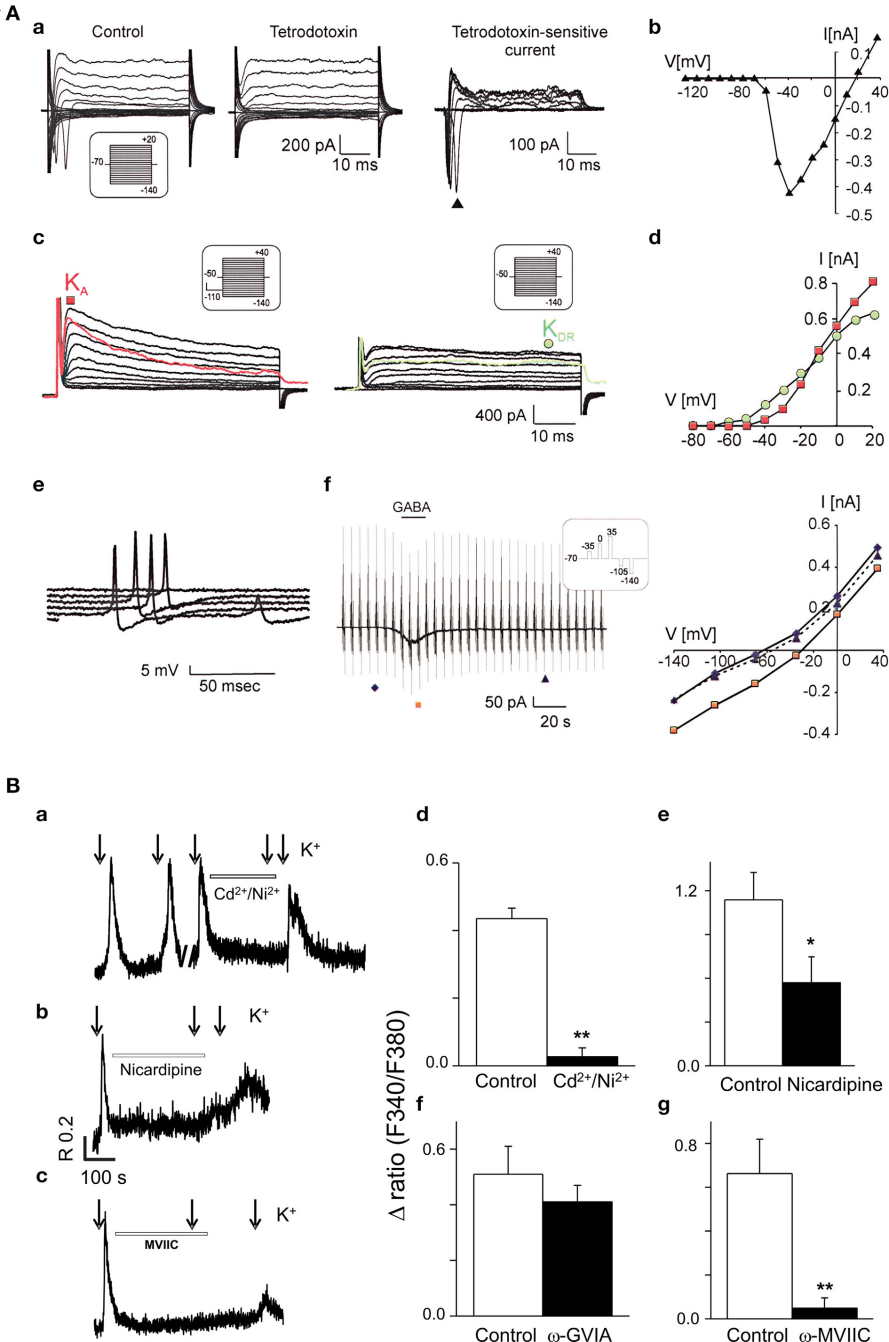
**Functional characterization of neurones derived from human stem cells. (A)** CCTL14 hESC-derived neural precursors express a typical neuronal current pattern *in vitro*. (a) Typical membrane current pattern of a β-III tubulin-positive cell before and after the application of 1 μM tetrodotoxin and the tetrodotoxin-sensitive current. (b) The corresponding I/V relationship. (c) Fast activating A-type K^+^ current (K_A_, red color) and delayed outwardly rectifying K^+^ current (K_DR_, green color). (d) Corresponding I/V relationships for K_DR_ (filled circles) and K_A_ (filled squares). (e) Action potential generation by β-III tubulin-positive cells demonstrated by representative voltage responses to an increasing current injection (in 10-pA steps). (f) GABA-evoked currents (left) and corresponding I/V relationships for control traces prior to (filled diamonds), during (filled squares), and after GABA washout (filled triangles, right). Reproduced from Kozubenko et al. (2010) with kind permission of Cognizant Communication Corporation (Putnam Valley, NY, USA). **(B)** Voltage-operated Ca^2+^ channels in CCTL14-P7 hESC-derived neural precursors. Examples of Fura-2 fluorescence traces (a–c) from individual cells for each experimental design showing the block of 50 mM K^+^ (applied 10 s, see arrows) responses by specific Ca^2+^ channel antagonists. The cells were first exposed to a control 50 mM K^+^-induced depolarization and the [Ca^2+^]_i_ responses were monitored. Subsequently, the same cell was pre-incubated for 5 min with Ca^2+^ channel blockers (Cd^2+^/Ni^2+^, non-selective high and low-VGCC; nicardipine, L-type blocker; ω-GVIA, N-type blocker and ω-MVIIC, P/Q-type blocker) as indicated on the trace and then again challenged with high K^+^. Pre-incubation of cells with 100 μM Cd^2+^ together with 50 μM Ni^2+^, nicardipine 10 μM and ω-conotoxin MVIIC (300 nM) significantly reduced the [Ca^2+^]_i_ responses (about 85, 54, and 92%, respectively; *n* = 5–9 for each group). No significant inhibition (only about 20%) was observed in the presence of N-type blocker, ω-conotoxin GVIA 800 nM (*P* = 0.27; *n* = 9). The bar diagrams (d–g) are cumulative data showing the reduction of high K^+^-induced [Ca^2+^]_i_ responses by L- and P/Q-type Ca^2+^ channel blockers (e and g). (^*^P < 0.05, or ^**^P < 0.001) vs. control K^+^ stimulus. Data and figure revised from Forostyak et al. (2013) with kind permission of Mary Ann Liebert, Inc., publishers (New Rochelle, NY, USA).

Patch-clamp experiments unveiled three main types of currents in the hESC-derived NPs: (i) tetrodotoxin-sensitive currents, indicative of functional voltage-gated Na^+^ channels, (ii) fast activating A-type K^+^ current and delayed outwardly rectifying K^+^ current, and (iii) GABA-evoked currents (Figure 2A). The resting membrane potential reached values of around −60 mV 5 weeks after the induction of differentiation (Kozubenko et al., 2010).

In addition, and as stipulated above, investigations based on the measurements of the intracellular calcium concentration ([Ca^2+^]_i_) in single cells showed and confirmed the expression of functional VGCCs (Figure 2B), intracellular RyRs, sarco-endoplasmic reticulum calcium-ATPase pumps, as well as glutamate and purinergic receptors. Moreover around 30% of the hESC-derived NPs (passage 7) displayed a series of spontaneous Ca^2+^ transients, referred to as oscillations, which were driven by extracellular Ca^2+^ and VGCCs (mainly L-type) (Forostyak et al., 2013).

Needless to mention, at this stage the full functional characterization of the CCTL14-derived NPs is an on-going process and further central points must be promptly addressed in order to better understand the remodeling of the Ca^2+^ homeostasis occurring during differentiation. Such central points include cell survival rate, the detailed quantification of the expressed receptors and channels, the expression of specific GABA and glutamate receptors, the role of inositol trisphosphate receptor-mediated signaling pathways, and the developmental profile of the resting [Ca^2+^]_i_.

A similar approach, conducted by our collaborators, was used to test the stem cell model—an immortalized neural stem cell line from human fetal spinal cord which preserves the features of ventral spinal cord progenitors even after extensive *in vitro* propagation and engraftment onto a lesioned rodent spinal cord (Cocks et al., 2013). From the cell lines generated, individual SPC-01-derived neurons exhibited similar Ca^2+^ signaling patterns to what was described previously in the case of CCTL14-derived NPs; particularly the presence of functional L- and P/Q-type Ca^2+^ channels and the occurrence of spontaneous Ca^2+^ oscillations.

## Transplantation assay

**Figure 3:**
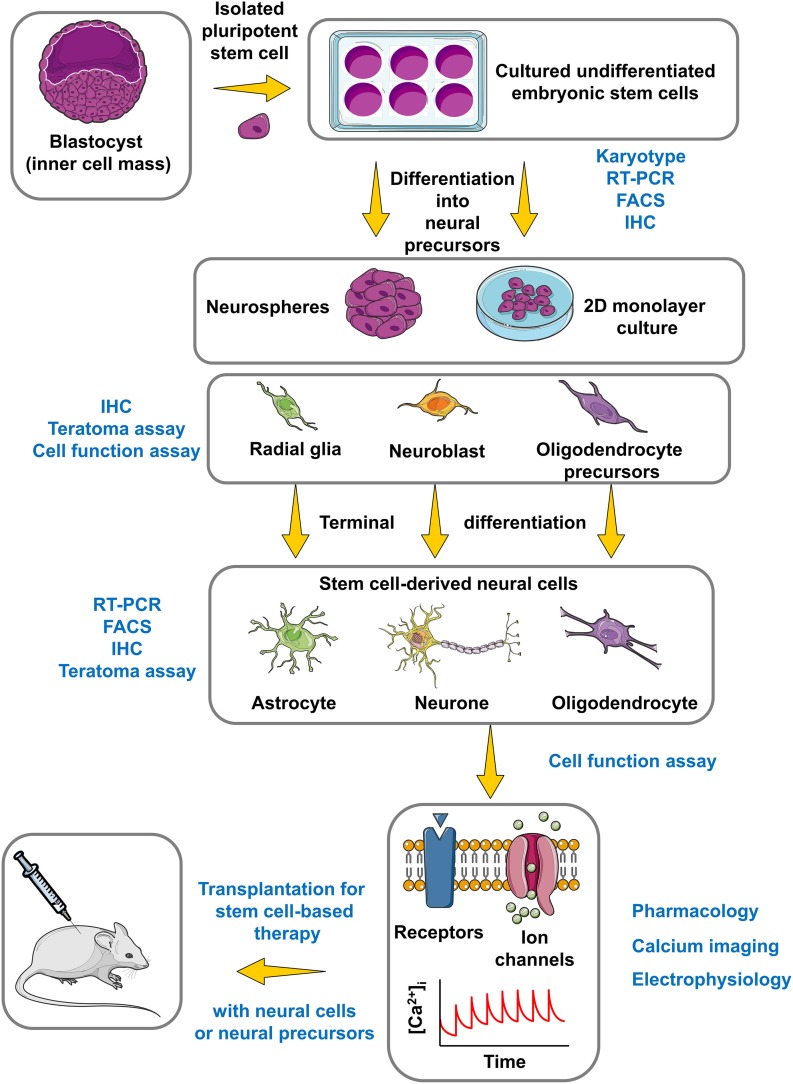
**Roadmap for the use of hESCs in CNS regenerative medicine**. This simplified and summarized flowchart illustrates the major important phases to obtain, differentiate and characterize a stem cell model with regenerative potential for the CNS regarding stem cell-based therapy. The experimental approaches of identification and characterization appear in blue color, while the protocol for stem cell culture and differentiation appears in black color. FACS, Fluorescence-activated cell sorting; IHC, Immunohistochemistry. The figure was produced using Servier Medical Art.

The last examination consisted of monitoring the survival and the differentiation of the hESC-NPs *in vivo*. For this purpose single cell suspensions were injected into the striatum of rat brains approximately 1 week after a stroke injury. The best results were obtained with NPs (passage 8 or P8) in terms of elevated survival, low tumor formation, and capacity of cell migration toward the lesion site (Kozubenko et al., 2010). These results were in the agreement with the data obtained from functional studies, showing that P7 and P8 were in the best physiological conditions among other passages. Furthermore the fact that attempts to engraft undifferentiated hESCs led to the formation of tumors in all cases serves as a teratoma assay and confirms the capacity of hESC pluripotency (Figure 3). Strikingly, prolonged periods of cell culturing decreased the quality of the NPs with regard to survival and migration. Finally it is worth mentioning that implantation of SPC-01 cells (a conditionally immortalized neural stem cell line derived from human fetal spinal cord tissue, see above) in Wistar rats resulted in distinct motor and sensory restoration after spinal cord injury (Amemori et al., 2013).

In neurodegenerative conditions such as Parkinson's and Alzheimer's diseases, engraftment experiments with stem cells also yielded promising results (Sykova and Forostyak, 2013). The transplantation of dopaminergic neurones derived from mouse ESC obtained either by fertilization or by nuclear transfer corrected the phenotype of mouse models of Parkinson's disease (Kim et al., 2002; Barberi et al., 2003). Moreover investigations employing human embryonic and transplanted neural stem cells provided useful information on the normal functions of Alzheimer's disease-related genes, their role in neural development, and therapeutic perspectives in animal models (Chen and Blurton-Jones, 2012).

## Conclusion

Here we summarized the information collected by our collaborators over the last few years in order to present an outline for the generation of a suitable human stem cell line for neural differentiation and further transplantation. The huge amount of data produced during the functional characterization of this cell line will serve as an invaluable basis to define a thorough profiling of the corresponding NPs. A unique ion channel and Ca^2+^ handling footprint will be assigned to each line of hESC-NPS. Thus cells, such as CCTL14-derived NPs, will participate in forming a pool of models for human cells that can be employed for basic and medical research, drug screening and cell therapy.

Beside the promising therapeutic strategies currently being tested in animal models, an increasing number of studies based on treatments of nervous system diseases by stem cells are entering clinical trials all over the world. Indeed companies, hospitals and universities have been approved to conduct Phase I clinical trials. It has been recently pointed out though that large discrepancies in the guidelines between the NIH Stem Cell registry and the FDA's requirements make the majority of hESC used not yet amenable to be tested in clinical trials (Jonlin, 2014). However, the first hESC-based treatment proposed by Advanced Cell Technology for Stargardt's Macular Dystrophy and Dry Age Related Macular Degeneration (Schwartz et al., 2012) has already been authorized by the FDA for Phase I/II clinical trials. In addition, Neuralstem, a company using human spinal cord derived stem cells (from fetus, not embryos) developed a therapy for amyotrophic lateral sclerosis which is currently being tested in Phase II clinical trials after approval by the FDA.

### Conflict of interest statement

The authors declare that the research was conducted in the absence of any commercial or financial relationships that could be construed as a potential conflict of interest.
